# Performance Medicine: a novel and needed paradigm for proactive health care

**DOI:** 10.3389/fspor.2023.1156645

**Published:** 2023-07-21

**Authors:** Richard J. Joseph, Rachele M. Pojednic

**Affiliations:** ^1^ Division of Preventive Medicine, Brigham and Women’s Hospital, Harvard Medical School, Boston, MA, United States; ^2^ Department of Health and Human Performance, Norwich University, Northfield, VT, United States; ^3^ Institute of Lifestyle Medicine, Harvard Medical School, Boston, MA, United States

**Keywords:** Performance, preventive health, Medicine, lifestyle, behavior

## Abstract

Performance Medicine is an emerging clinical practice that holds immense promise for advancing preventive health. To date, however, the concept remains imprecise, disorganized, and commercialized. The purpose of this perspective article is to define characteristics, core tenets, and practice standards to help build a common framework. We define performance broadly as “one’s capacity to bring energy and attention to what matters most in a given moment”. Performance Medicine, therefore, is predicated on the thesis that the critical practices that enhance one's daily wellbeing simultaneously increase both lifespan and healthspan. As a clinical practice, Performance Medicine is proactive and preventive. It focuses on the immediate and actionable strategies to address one's physical, mental, and emotional capabilities every day. The practice employs a values-centered approach that begins with a discovery process to elucidate the client's deeply held beliefs about their health status, life mission and goals, vision for optimal wellbeing, and motivations for change. Subsequent diagnostics and therapies combine evidence-based practices from multiple medical specialties including internal medicine, sports medicine, obesity medicine, integrative medicine, and others. This is complemented by the most recent scientific advancements in nutrition, exercise physiology, sleep, and recovery. The Performance Medicine prescription incorporates a personalized combination of lifestyle-based behavior change practices, evidence-based diagnostics and risk reduction therapies, ongoing monitoring, and community support. Finally, the iterative and incremental process towards enhanced and sustained health is guided and supported by a trusted partnership between the client and a team of expert practitioners and coaches.

## 1. Introduction: the current medical model—disappointing and deficient

The U.S. spends over 4 trillion dollars–nearly one fifth of our nation's entire wealth—on health care each year. For $12,000—the average annual cost per person—the typical American can expect to live 77 years. This life expectancy is five years less than counterparts from comparable industrialized countries, which notably spend far less on health care (1). Moreover, this projection does not consider the quality of those 77 years. Recent data demonstrate the burden of disease in the United States is led by growing prevalence of “deaths of despair”—death by drugs, alcohol and suicide—and a slowdown in progress against leading causes of mortality like heart disease and cancer (2). The COVID-19 pandemic further increased mortality (3) and premature death rates (4) in the U.S. by more than it did in most peer countries.

The data is not only deplorable; it is downright damning.

The World Health Organization defines health as, “a state of complete physical, mental and social well-being and not merely the absence of disease or infirmity” (5). Yet the current medical system remains problem oriented, disjointed, and reactive to illness and injury. Amidst ongoing calls for preventive strategies in medicine and public health (1), little change is occurring in the traditional model of care. Providers lack time, training, and team to effectively deliver personalized lifestyle-based therapies (6) that have repeatedly been shown to maintain health and enhance wellbeing. Furthermore, the fee-for-service reimbursement structure, still the predominant healthcare financing model, values quantity of services rather than quality of health itself (7).

Critical years, when individual health behaviors crystalize and when high performance is essential for long-term health, often receive the least expert attention (8). Individuals aged 19–50 years old typically see a primary care once per year, or less, and care is most often reactive to early stages of disease and dysfunction (8). Moreover, a decreasing proportion of Americans have reported an identified source of primary care, especially Americans who were younger, less medically complex, of minority background, or living in the South (9). This lack of attentiveness to the upstream determinants of health (10) increases the risk of disease and dysfunction to insidiously take root. As such, we engage healthcare only later in life when disease prevention cedes ground to disease management (1).

Dissatisfaction with the current medical system is understandably high, with those experiencing more barriers to preventive self-care prior to disease diagnosis perceiving the most frustration (11). If a patient does not meet health insurance criteria for disease, traditional primary care has little to offer, with the utility of the annual checkup suspect at best (12). Rarely does routine care provide an actionable plan, behavioral strategies, or guidance for how to maintain or improve health. Instead, most patients are left to cobble together a patchwork of health solutions in the wild west of “wellness” where gimmicks, snake oil, and fads abound. The lack of standards, dogmatic ideologies, and extreme approaches within the wellness and fitness industries present not only a challenge, but a potential harm to the individual (7). A recent call to action noted that “pseudoscience and so-called “quick fix’ interventions undermine initiatives aimed at evoking long-term behavior change, impede the ongoing pursuit of…performance, and lead to serious downstream consequences for clinical practice” (13).

Put together, there is a gaping chasm where a preventive and proactive healthcare model should exist. Since 1954, the field of preventive medicine has tried to address upstream causes of disease, with its explicit goal of “preventing disease, disability, and death,” (14). However, the field has fallen short, largely due to a dire lack of representation in the medical specialties. Preventive medicine physicians currently make up less than 0.8% of the physician workforce and federal funding provides only 26 residency slots in five preventive medicine programs nationwide (15). Moreover, the core disciplines of preventive medicine and public health are biostatistics, epidemiology, health policy and administration, health behavior and environmental health (14, 15). Although vaccinations and screenings are lifesaving, even preventive medicine physicians are not trained to address the behaviors that contribute to the most prevalent conditions in the United States.

Moreover, the very concept of prevention—avoiding some hypothetical disease in the distant future—has never been particularly pertinent, persuasive, or pleasurable to most people (16). An estimated nine in ten American adults are considered metabolically unhealthy (17) and six in ten have at least one chronic illness, with nearly half suffering from multiple chronic illnesses (18). Approximately 80% of chronic disease diagnoses can be prevented, mitigated, and even reversed by participating in four healthy behaviors: never smoking, having a body mass index lower than 30 kg/m^2^, performing 3.5 h/wk or more of physical activity, and adhering to healthy dietary principles (19). And yet, less than 3% of the American population engage in all four of these behaviors (20) and the majority physicians are not trained in the lifestyle tenets essential for optimal health—proper nutrition, weight management, physical activity and behavior change (6, 19).

Amid a global pandemic and increased awareness of our nation's ubiquitous health inequities, we are necessarily reconsidering what it means to be healthy and what we need from our healthcare (21). The current medical model is woefully deficient. It is not designed to deliver health and wellbeing. We need a paradigm shift. We need Performance Medicine.

## 2. Performance Medicine

### 2.1. A new and necessary paradigm

Performance Medicine is an emerging ethos and clinical practice that holds immense promise. We define performance broadly as “one's capacity to bring energy and attention to what matters most in a given moment”. Performance Medicine is predicated on the thesis that the critical practices that enhance one's daily wellbeing and performance simultaneously increase the odds of both a long healthspan–the period of life that one remains healthy–and lifespan–the total duration of one's life.

**Figure 1:**
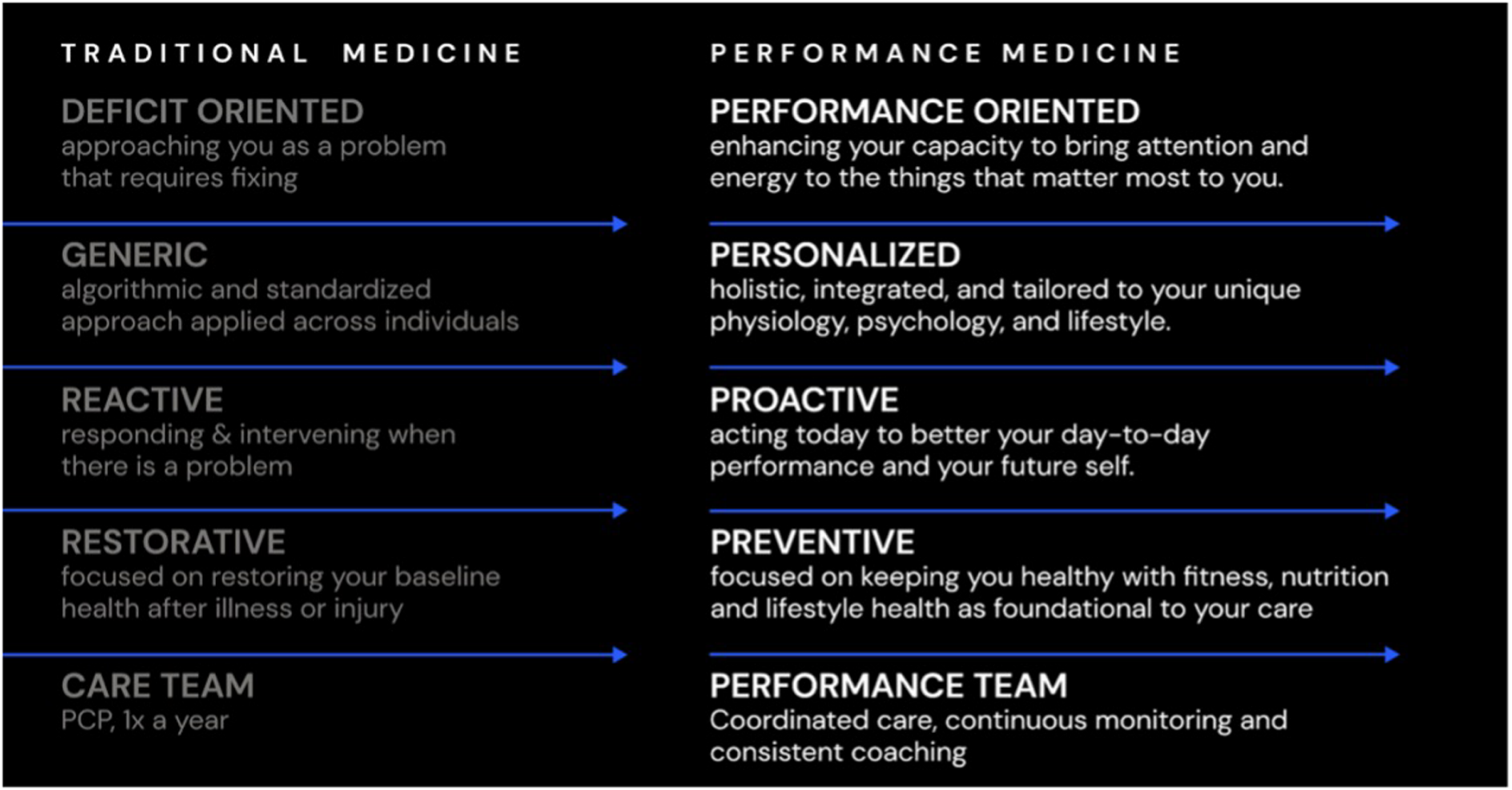
Performance Medicine is new paradigm for proactive health care. In contrast to the current medical model, Performance Medicine is personalized, proactive and preventive by focusing on increasing performance across the physical, mental, and emotional domains of daily functioning.

To date, however, the concept remains imprecise, disorganized, and commercialized. As such, we propose defining characteristics, core tenets, and practice standards to help build a common framework (Figure 1).

Outlining what Performance Medicine is begins by specifying what it is not. Performance Medicine is not a dogmatic ideology or rigid care model, a one-size fits all approach, a templated therapy or specific prescription. Rather, it is a philosophy and perspective on human health that informs a proactive design and delivery of care. In the context of our current healthcare delivery system, Performance Medicine is a new paradigm which is especially salient for individuals who are currently at low risk of disease, in remission, or perhaps managing chronic illness but in need of a more longitudinal risk reduction and health promotion strategy. This large segment of the population does not require (or want) traditional disease-based medical care (11), but needs the proper tactics, skills, and support to improve their immediate daily performance while sustaining long-term health. Performance Medicine can be thought of as an antecedent to disease based care, with a goal of preventing the root and progression of the most prevalent diseases and causes of death of our time (22).

Performance Medicine distinguishes itself from other non-traditional models [i.e., Lifestyle Medicine (23), Integrative Medicine (24), or Functional Medicine (25)] in that it is not a reactive post-diagnosis treatment strategy utilizing alternative, complementary, or behavioral therapies in place of usual care. Instead, Performance Medicine proactively seeks to keep well people well. It is a new paradigm that is designed to fill the gaping chasm where preventive and proactive healthcare is currently missing. Whereas the traditional medical model is satisfied with patient survival, Performance Medicine encourages daily human thriving. Although progressive, Performance Medicine is also firmly founded in evidence-informed practices, led by experts in their fields, which clearly delineates and elevates the model above wellness influencers and businesses utilizing misappropriated claims simply for financial gain (13).

Before proceeding, it is critical to acknowledge that for those struggling to make ends meet, those encountering adverse environments and circumstances, those afflicted by injustice and trauma—the very act of survival is a daily, and often herculean challenge. The notion of thriving might appear unattainable and, therefore, prohibitive and even off-putting. However, we maintain and steadfastly believe that every individual, regardless of socioeconomic status, race and ethnicity, educational background, and life circumstance is entitled to the pursuit of, and realized performance. The process of seeking health confers values like self-respect, courage, and fortitude. Consistent and deliberative acts to protect and preserve our wellness are, in effect, statements that our lives have meaning. We are worthy of a vigorous and vibrant existence. Performance Medicine is predicated on the foundational belief that all humans have a right to health and agency to improve their quality of life.

### 2.2. Clinical aims and objectives

As a clinical practice, Performance Medicine focuses on the immediate and actionable imperative of improving and maintaining one's physical, mental, and emotional performance every day. The practice employs a values-centered approach that begins with a discovery process to elucidate the client's deeply held beliefs about their health status, life mission and goals, vision for optimal performance, and motivations for change. The diagnostics and therapies combine evidence-based practices from multiple medical specialties including internal medicine, sports medicine, obesity medicine, integrative medicine, and others. This is complemented by incorporating the most recent scientific advancements in nutrition, exercise physiology, sleep, and recovery. The Performance Medicine prescription incorporates a personalized combination of lifestyle-based behavior change practices, evidence-based diagnostics and risk reduction therapies, ongoing monitoring, and community support. And the iterative and incremental process towards enhanced health and performance is guided and supported by a trusted partnership between the client and a team of expert practitioners and coaches.

The clinical objectives of Performance Medicine are threefold:

1. To increase the individual's adaptive capacity throughout life with personalized training of the mind and body.
2. To employ behaviors and therapies that increase the individual's likelihood of postponing physical decline, preventing disease, and protecting against injury.
3. To instill a sense of agency and wellbeing that positions the individual to contribute to the betterment of society.

### 2.3. Performance Medicine in practice

Performance Medicine can be creatively delivered within myriad models—multiple practitioner stand-alone clinics, multi-location referral networks within and between clinical and wellness systems, or by a single clinician with a well curated network of external collaborators. Indeed, these types of models have be used to deliver patient-centered lifestyle related programs such as Exercise is Medicine (26), Exercise Oncology (27) or the Diabetes Prevention Program (28).

Our team currently delivers Performance Medicine through a medically supervised, virtual first, performance-coach led service. The core pillars of the care model include a thorough intake to fully detail a client's unique physiology, psychology, and personal preferences; an actionable Performance Plan designed by the integrated performance team; ongoing coaching and accountability; interval monitoring and testing; and access to a network of specialist practitioners. We include several highly educated practitioners in our model of care, trained in key topics related to behavior change and cognitive behavioral coaching (29). This strategy provides assurance to clients that their individualized needs are being met, across several specialties and lifestyle interventions, in an evidence-informed manner.

To effectively deliver our Performance Medicine model, our care team includes:

1. Performance Physicians: Medically trained practitioners who provide preventive health services, testing, and prescription therapies. The Performance Physicians oversee and integrate care amongst other practitioners/specialists and communicate with providers in the healthcare delivery system.
2. Performance Coaches: Experts in behavior change who have nationally accredited training in health coaching, exercise and/or nutrition science. These certifications could include the National Board of Health and Wellness Coaches Health and (NBHWC NBC-HWC) (30), American College of Sports Medicine Certified Exercise Physiology (ACSM-EP) (31) or Clinical Exercise Physiology (ACSM-CEP) (32) and/or a Registered Dietitian (RD/RDN) (33). The Performance Coaches provide clients with evidence-based instruction, expert coaching, and ongoing accountability.
3. Community Director: Expert in group dynamics who orchestrates and coordinates community events, manages virtual forums and targeted content delivery, & facilitates connections among clients. This way, health can become less of a solitary pursuit and more of a team sport.

We believe building better health and improving performance is not a passive process that can be outsourced or automated; it requires investment of time and energy. Participation in our Performance Medicine practice demands an individual's active engagement. While each client's motivation is certainly unique (34), clients all intentionally subject themselves to varying types and doses of stimuli in order to expand what is known as adaptive capacity—the ability of a person and their physiological systems to manage internal or external perturbations, i.e., to turn stressors into growth opportunities (35) to better manage the inherent challenges of life.

A ready example is using the stimulus of strenuous exercise to increase physical fitness. We believe physical activity, in particular, is the best preventive and restorative medicine and the most potent protector against disability, disease, and death (36). Time and again, we have seen it act as a powerful gateway behavior and keystone habit (37) for people to become more interested, engaged, and committed to their health. And yet, less than a quarter of US adults participate in regular physical activity, with many leading completely sedentary lifestyles (38). To make matters worse, education about the physical and mental benefits of physical activity in healthcare professions is sorely lacking (39). And health & fitness professionals remain disconnected from the traditional health care delivery system without mechanisms for referrals, interprofessional training, or standards of care (39).

**Figure 2:**
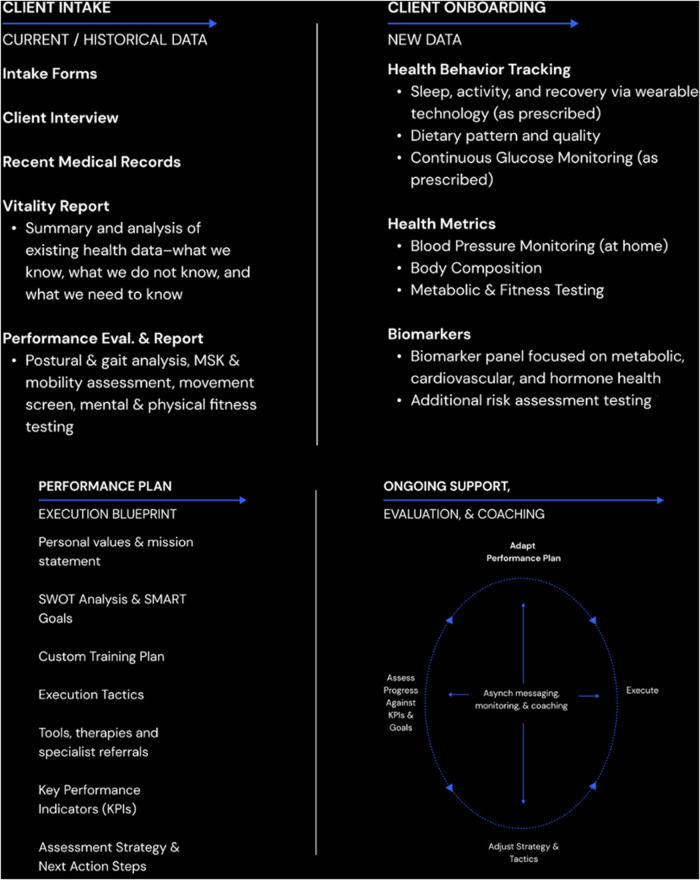
The client care journey in a representative performance medicine model.

Our model seeks to make physical activity a core pillar of care and each client receives a fitness plan that includes multimodal exercise, a custom strength training plan, and personal training support. We use physical training as not only the primary prescription to improve health and physical performance, but as the medium to explore adaptive capacity in other domains of life and an approach to self-development that prioritizes patience, incremental progress, and process over outcomes (Figure 2).

### 2.4. Addressing access and equity

At its core, Performance Medicine is about using the pursuit of health and wellbeing as a mechanism for increasing one's sense of agency and empowerment for life more broadly. Performance Medicine can and must be accessible to any and everyone who wants to partake in both the philosophy and the practice. We believe that implementing proactive and upstream approaches not only improve lifespan and healthspan for all, but are critically important to addressing health related inequities and disparities (10).

That said, we fully appreciate how aspirational ideals like self-development, thriving, and even health itself are necessarily deprioritized when competing against more immediate survival needs–food, shelter, safety, and a stable wage (40). We live in a nation that, despite its riches, does not guarantee the right to health for all of its citizens. The nation's healthcare delivery system operates on financial incentives that reward sickness over health.

Given this regrettable reality, Performance Medicine necessarily begins as an elective clinical service for a demographic with the ability and willingness to pay for care. Due to a lack of reimbursement for preventive behavioral care, most Performance Medicine practices will likely opt out of an insurance-based model. However, the field of Performance Medicine must prioritize the democratization of the model across all socioeconomic groups. And this process of increasing access and equity in Performance Medicine must start now, while the field is in its infancy.

To begin, practices can offer highly discounted group-based Performance Medicine programs that provide education, peer support, and connections to community-based wellness and healthcare resources in nearby community spaces. A portion of the revenue generated in Performance Medicine practices can be used to support the operations of partner non-profits working to achieve health and health equity for all. As the care model becomes more widespread and standardized, Performance Medicine practice networks and professional societies must push for insurance coverage for selective services and test income-based fee-for-service models that subsidizes services for those in lower income brackets. In addition, Performance Medicine care models must work to build the operational efficiencies and supportive technologies that will drive down the price point over time. The business model to support a Performance Medicine care model must start somewhere, but it must not stop in its relentless pursuit of equity.

## 3. Performance Medicine: looking forward

In a nation of disgruntled patients, disillusioned providers, and deficient healthcare, we introduce Performance Medicine as an aspirational approach, an inclusive ethos, and a viable care model to health and wellbeing that will prompt a more proactive paradigm of health care. While establishing Performance Medicine as a pillar of accepted clinical practice is a long-term goal, we believe defining the parameters of the model is an essential first step. We discuss the core components of a representative Performance Medicine clinic and posit how such delivery models achieve financial sustainability while simultaneously advancing access and equity. However, we also recognize that there will be barriers to implementation, which include training and reimbursement of expert practitioners, the current lack of structures and resources for this model at every level of healthcare, and the overall cost. Solutions will require integration and input from sectors across the fields of health promotion, and shared experiences and approaches will be critical as Performance Medicine establishes a foothold. Ultimately, we encourage practitioners to design, and health-conscious clients to seek, similar care models that leverage the tenants of Performance Medicine with the belief that together, we can all achieve healthier, more vital lives.

## Data Availability

The original contributions presented in the study are included in the article/Supplementary Material, further inquiries can be directed to the corresponding author.
